# Effects of Particle Size on the Dielectric, Mechanical, and Thermal Properties of Recycled Borosilicate Glass-Filled PTFE Microwave Substrates

**DOI:** 10.3390/polym13152449

**Published:** 2021-07-26

**Authors:** Ibrahim Abubakar Alhaji, Zulkifly Abbas, Mohd Hafiz Mohd Zaid, Ahmad Mamoun Khamis

**Affiliations:** 1 Department of Physics, Faculty of Science, Universiti Putra Malaysia, Serdang 43400, Malaysia; gs54099@student.upm.edu.my (I.A.A.); mhmzaid@upm.edu.my (M.H.M.Z.); akhameis@yahoo.com (A.M.K.); 2 Department of Physics, Faculty of Science, Federal University of Kashere, Gombe PMB 0182, Gombe State, Nigeria; 3 Institute of Tropical Forestry and Forest Product, Universiti Putra Malaysia, Serdang 43400, Malaysia; 4 Institute of Advanced Technology, Universiti Putra Malaysia, Serdang 43400, Malaysia

**Keywords:** recycled borosilicate, PTFE, sintering, permittivity, high-frequency, substrates

## Abstract

Low dielectric loss and low-cost recycled borosilicate (BRS) glass-reinforced polytetrafluoroethylene (PTFE) composites were fabricated for microwave substrate applications. The composites were prepared through a dry powder processing technique by dispersing different micron sizes (25 µm, 45 µm, 63 µm, 90 µm, and 106 µm) of the recycled BRS filler in the PTFE matrix. The effect of the filler sizes on the composites’ thermal, mechanical, and dielectric properties was studied. The dielectric properties of the composites were characterised in the frequency range of 1–12 GHz using an open-ended coaxial probe (OCP) connected to a vector network analyser (VNA). XRD patterns confirmed the phase formation of PTFE and recycled BRS glass. The scanning electron microscope also showed good filler dispersion at larger filler particle sizes. In addition, the composites’ coefficient of thermal expansion and tensile strength decreased from 12.93 MPa and 64.86 ppm/°C to 7.12 MPa and 55.77 ppm/°C when the filler size is reduced from 106 μm to 25 μm. However, moisture absorption and density of the composites increased from 0.01% and 2.17 g/cm^3^ to 0.04% and 2.21 g/cm^3^. The decrement in filler size from 106 μm to 25 μm also increased the mean dielectric constant and loss tangent of the composites from 2.07 and 0.0010 to 2.18 and 0.0011, respectively, while it reduced the mean signal transmission speed from 2.088 × 10^8^ m/s to 2.031 × 10^8^ m/s. The presented results showed that PTFE/recycled BRS composite exhibited comparable characteristics with commercial high-frequency laminates.

## 1. Introduction

The last decade has seen rapid and unprecedented developments in information technology driven by military and consumer markets [1,2,3]. This change creates demands for high-speed, light and low-cost microwave substrate. A microwave substrate that meets specific criteria supports microwave circuits [4,5,6]. Microwave substrates are dielectric materials with low permittivity and a low loss tangent at microwave frequencies [5]. The substrate materials should have the following properties: low permittivity and loss tangent for rapid signal propagation, low coefficient of thermal expansion (CTE) for dimensional stability, high thermal conductivity for transporting the heat generated away from the microwave circuit and good mechanical strength for material rigidity [7].

Polymers are employed for substrate applications due to their excellent electrical properties. Polytetrafluoroethylene (PTFE) is the most widely used among polymers because of its low permittivity, dielectric loss, moisture absorption and chemical inertness [8,9,10]. However, it has a high CTE (~109 ppm/°C) and melting point (~327 °C) that hinder its utilisation [11]. It also lacks rigidity for practical substrate applications. These limitations can be overcome by adding inorganic and rigid fillers such as glass with lower CTE and moderate dielectric properties. That is possible because the properties of polymers depend on their microstructure and composition [12]. The high melting point of PTFE can also be circumvented by employing a processing technique, such as the powder processing method, that does not require heat treatment to mix PTFE-glass composites [13].

Recently, recycled glass fillers have attracted considerable attention for microwave applications due to their rigidity and moderate dielectric properties [14]. Recycled glass is cheaper and reduces environmental pollution. In this work, the preparation and characterisation of recycled borosilicate glass filled PTFE substrate is reported. Borosilicate (BRS) is an industrial glass with a thermal conductivity ranging from 1–1.3 W/mK. It has a low CTE of 3.2 ppm/°C–4.0 ppm/°C and tensile strength of about 22 MPa–32 MPa. The glass is also an excellent electrical insulator with a dielectric constant and loss factor of 4.65–6.00 and 0.01–0.017 [15,16]. These excellent properties of BRS glass make it a perfect filler when recycled for PTFE-based substrate applications. To the best of our knowledge, no systematic study of the effect of the recycled BRS filler size on PTFE/recycled BRS composites has been reported. Therefore, this work investigated the dielectric, thermal, and mechanical properties of PTFE/recycled BRS. In addition, signal propagation speed across the composites with different filler sizes was calculated and analysed. The PTFE/recycled BRS composite was also compared with commercial high-frequency laminates.

## 2. Materials and Methods

### 2.1. Materials

The PTFE of type MF90C with an average particle size of 50–110 μm was obtained from Fujian Sannong New Materials Co., Ltd., Sanming, China. At the same time, BRS glass was acquired from Top Globe Sdn. Bhd. Selangor, Malaysia, in the form of waste moulds.

Glass Powder Preparation

The BRS glass moulds were initially cleaned, washed, and dried at room temperature for 24 h. After that, the moulds were crushed with a hammer into glass pebbles. A Plunger was further used to grind the glass pebbles into coarse glass powder. In addition, the coarse glass powder was transferred to a grinding mill jar with a powder-to-ball ratio of 20:1, which was then milled. The milling was conducted at room temperature for 24 h at 45 rpm using the U.S. Stoneware Jar Mills (U.S. Stoneware, East Palestine, OH, USA). After the milling stage, the recycled BRS powder was sieved to 25 µm, 45 µm, 63 µm, 90 µm, and 106 µm particle sizes. The range of these representative filler particle sizes is given in Table 1.

### 2.2. Preparation of PTFE/Recycled BRS Composites

The PTFE/recycled BRS composites were prepared by mixing 25 µm, 45 µm, 63 µm, 90 µm, and 106 µm of the recycled BRS filler with PTFE through a dry powder processing technique. The mixing was conducted via a Wing dry mixer for 10 min, and filler content in each composite was fixed at 5 wt.%. Then, the compositions were pressed into preforms using a hydraulic press at a pressure of 10 MPa for 5 min. The compacted composites were mechanically weak due to air voids. Hence, sintering is required for the removal of the voids. The samples were sintered from room temperature to 380 °C with a temperature rising time of 3 °C/min and held for 1 h to allow for particles fusion, coalescence and void elimination in the composites. The cooling rate was set at 1 °C/min from 380 °C to room temperature to complete the sintering cycle. A Drying Oven (Jiangsu Sunkoo Machine Tech Co., Ltd., Changzhou, China) was utilised for the sintering.

### 2.3. Characterisations

#### 2.3.1. Phase, Morphology and Composition

In this work, XRD was employed to analyse the phase formation of recycled BRS powder and PTFE/recycled BRS composites. The XRD data were collected using an automated Philips X’pert system (Model PW3040/60 MPD) with Cu–Kα radiation operating at a voltage of 40.0 kV and a current of 40.0 mA with a wavelength of 1.5405 Å. The 2-theta range of 10°–70° with a scanning speed of 2.0 °/min was used to record the diffraction patterns. All data were exposed to the Rietveld analysis on X’Pert Highscore Plus v3.0 software (PANalytical B.V., Almelo, The Netherlands). The samples were classified by comparing their diffraction peaks with the Inorganic Crystal Structure Database (ICSD).

The shape, arrangement and dispersion of the recycled BRS particles in the composites were investigated using LEO 1455 Variable Pressure Scanning Electron Microscope (VPSEM, Leo Electron Microscopy Group, Oberkochen, Germany). The elemental composition of the samples was obtained via an Oxford Inca energy dispersive X-ray micro-analyser (EDX, Oxford Instruments, Buckinghamshire, England) attached to the Leo 1455 VPSEM. Five spots on each sample were examined with the EDX for accurate determination of the elemental compositions of the composites qualitatively.

#### 2.3.2. Moisture Absorption

The presence of moisture within a material increases its dielectric properties [17]. This change degrades the performance of the materials. Thus, determining the moisture absorption of materials is essential to identify suitable environmental operating conditions. PTFE/recycled BRS composites were cut into 25.4 mm by 76.2 mm following the ASTM D570 standard. The samples were then immersed in distilled water at 25 °C for 24 h. The percentage of moisture absorption for the composites was calculated according to Equation (1) [18].

(1) $$MA\;\left(\%\right)=\frac{w_{f-}w_{i}}{w_{i}}\times 100$$

where $w_{f}$ and $w_{i}$ are the respective wet and dry weights of the samples.

#### 2.3.3. Density

The density of the PTFE/recycled BRS composites was measured at room temperature using the Archimedes principle. An electronic densitometer (Alfa Mirage Co., Osaka, Japan) was utilised for the measurement. Distilled water was then used as the reference liquid. Hence, the density of the sample was calculated using the following equation [19].

(2) $$\rho_{c}=\frac{W_{air}}{W_{air}-W_{distilled\;water}}\times \rho_{distilled\;water}$$

where $\rho_{c}$ is the density of the composite, $\rho_{distilled\;water}$ is the density of distilled water, and $w_{air}$ and $w_{distilled\;water}$ are the weights of the sample in air and distilled water, respectively.

#### 2.3.4. Tensile Strength

The dimensions of PTFE/recycled BRS composites were cut according to the ASTM D638 to determine the tensile strength of the composites [20]. The tensile strength test was conducted at room temperature using a Shimadzu AGS-X 100 kN computerised universal testing machine (UTM, Shimadzu, Kyoto, Japan). The UTM stretched the samples at a 5 mm/min stroke rate with a 10 kN load cell.

#### 2.3.5. Coefficient of Thermal Expansion (CTE)

The CTE of the composites was measured in line with ASTM E228-17 [21]. A push-rod dilatometer, Linseis L75 Platinum (Linseis, Selb, Germany), was used. The measurement was done at room temperature, and the heating rate was set at 10 °C/min.

#### 2.3.6. Complex Permittivity

**Figure 1:**
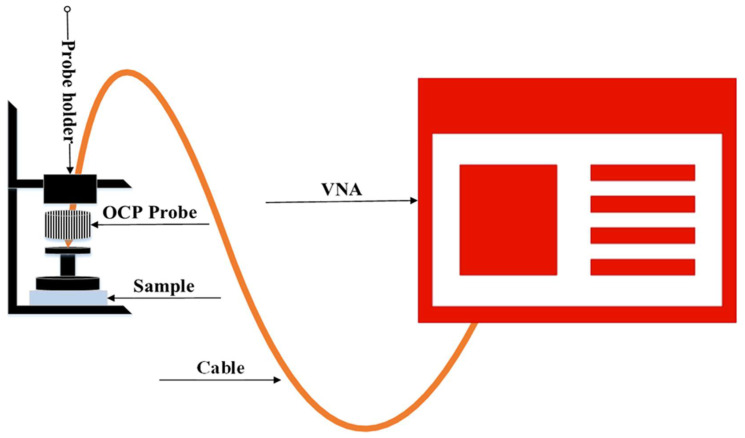
OCP measurement set-up.

The complex permittivity of PTFE/recycled BRS composites was characterised using the open-ended coaxial probe (OCP) technique in the 1–12 GHz frequency range [22]. The probe was connected to an Agilent N5227A vector network analyser (Agilent Technologies, Santa Clara, CA, USA), as shown in Figure 1. A one-port reflection calibration technique was used. The one-port calibration technique consists of air, a shorting block and distilled water at 25 °C. After complete calibration, the probe was placed flat on the surface of the samples for characterisation to avoid air gaps between the sample and the open probe that may affect measurement accuracy. A standard (unfilled PTFE) material was first characterised to confirm the accuracy of the calibration. In addition, the dimensions of the composites were 6 cm × 3.6 cm × 0.7 cm. 

The following equation gives the complex permittivity:

(3) $$\varepsilon^{*}=\varepsilon^{\prime }-j\varepsilon^{\prime \prime }$$

where $\varepsilon^{*}$ is the complex permittivity, $\varepsilon^{\prime }$ is the dielectric constant denoting energy storage, and $\varepsilon^{\prime \prime }$ is the loss factor, representing energy loss. The loss tangent, being the ratio of loss factor and dielectric constant, is therefore evaluated as follows [23]:

(4) $$tan\delta =\frac{\varepsilon^{\prime \prime }}{\varepsilon^{\prime }}$$

#### 2.3.7. Signal Propagation Speed

A fast signal transmission with minor delay is required to transmit high data. Generally, electromagnetic waves are attenuated when passing through a denser medium. Thus, investigating the influence of filler size on the signal propagation speed is critical to the design of microwave circuits for efficient data transmission. The signal transmission speed can be calculated using the following equation [24].

(5) $$V_{s}=\frac{c}{\sqrt{\varepsilon^{\prime }\mu^{\prime }}}$$

where $V_{s}$ is the signal transmission speed, *c* is the speed of light in vacuum, $\varepsilon^{\prime }$ is the dielectric constant, and $\mu^{\prime }$ is the permeability of the material.

## 3. Results and Discussion

### 3.1. Phase, Morphology and Composition

**Figure 2:**
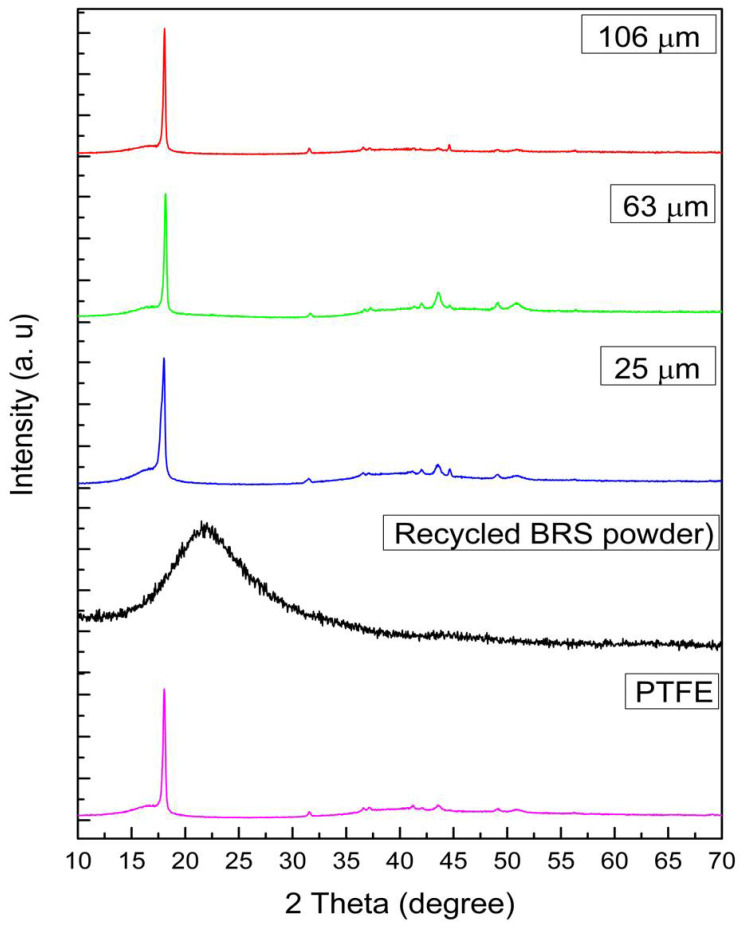
XRD patterns of PTFE, recycled BRS powder and PTFE/recycled BRS composites.

The X-ray diffraction patterns of 63 µm recycled BRS powder and PTFE/recycled composites are shown in Figure 2. In the 63 µm recycled BRS XRD profile, a broad peak at 2θ=15°−30° is observed, confirming the amorphous nature of the recycled BRS glass. This pattern is consistent with the work presented [25], which affirms that no impurities were introduced during the glass powder preparation. The same figure depicts the XRD pattern of PTFE. The diffractogram of the PTFE displays a sharp peak and five low-intensity peaks positioned at 2θ = 18.05°, 31.53°, 36.60° 37.13°, 41.18°, and 49.07°. These peaks relate to the (100), (110), (200), (107), (108), and (210) planes and are matched with the ICSD index of PTFE (ICSD 00-047-2217) [26,27]. Furthermore, the intensity of the peak located at 2θ = 18.05° can be seen to decrease slightly as different sizes of recycled BRS filler are introduced to the PTFE matrix. In addition, no unwanted peaks in the pattern of the composites indicate that chemical interaction did not occur between the PTFE matrix and recycled BRS particulate.

**Figure 3:**
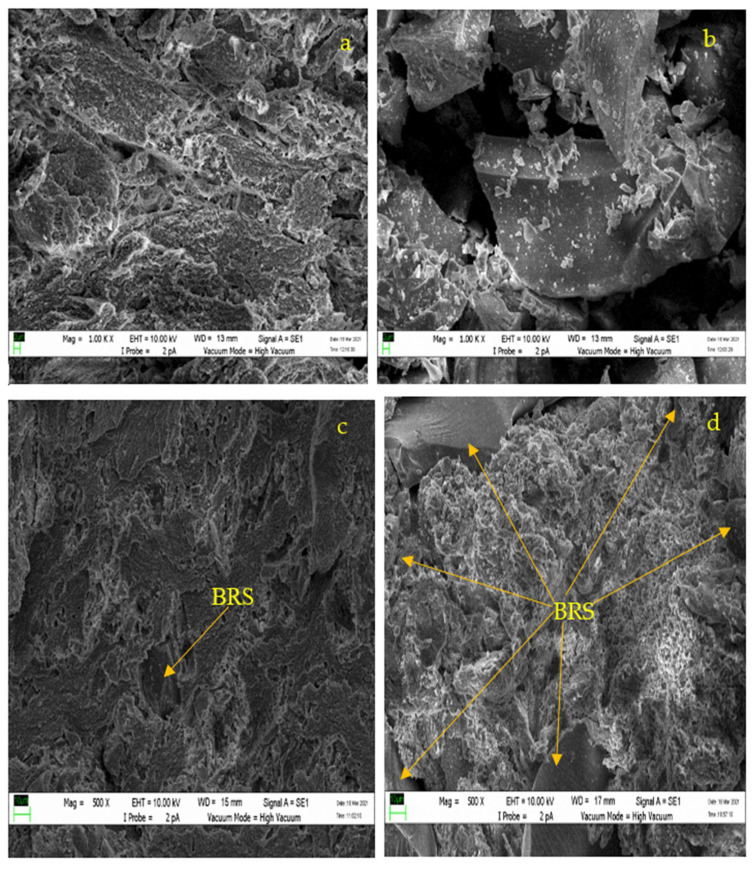
SEM micrographs of (**a**) PTFE, (**b**) recycled BRS powder, (**c**) PTFE/recycled BRS at (at 25 μm BRS) and (**d**) PTFE/recycled BRS composites at (106 μm BRS).

The scanning electron microscope (SEM) images of pure PTFE, 63 µm recycled BRS powder, and PTFE/recycled BRS composites are illustrated in Figure 3. It can be observed that the BRS particles are of arbitrary geometry. The recycled BRS particulates are also more dispersed in the PTFE matrix at larger filler sizes, indicating a good connection between the PTFE matrix and recycled BRS filler. It is reported that effective dispersion of recycled BRS particulate in the PTFE promotes a homogeneous structure that enhances the properties of the composites [28,29].

**Figure 4:**
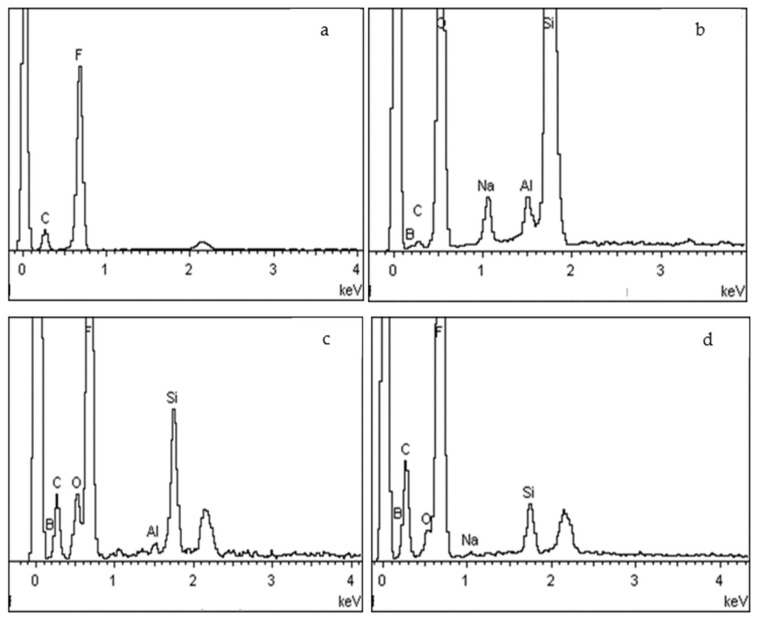
EDX spectra of (**a**) PTFE, (**b**) 63 recycled BRS powder, (**c**) PTFE/recycled BRS at 25 μm BRS and (**d**) PTFE/recycled BRS at 106 μm BRS.

EDX analysis was conducted to determine the elemental composition of PTFE, 63 μm recycled BRS and PTFE/recycled BRS composites qualitatively. In Figure 4, the spectra show that PTFE comprises mainly C at 0.1 keV and F at 0.5 keV. In addition, the same figure reveals that the 63 μm recycled BRS powder consists of B, O, Na, Al and Si, validating the purity of recycled BRS glass [30]. Further analysis shows that PTFE and recycled BRS glass elements were all present in the PTFE/recycled BRS composites except Na and Al at 25 μm and 106 μm recycled BRS filler loadings. This incidence happens when the concentration level of the respective element falls below the detection limit [31]. Thus, the findings attest to the suitability of the dry powder-processing technique for composite fabrication.

### 3.2. Moisture Absorption

**Figure 5:**
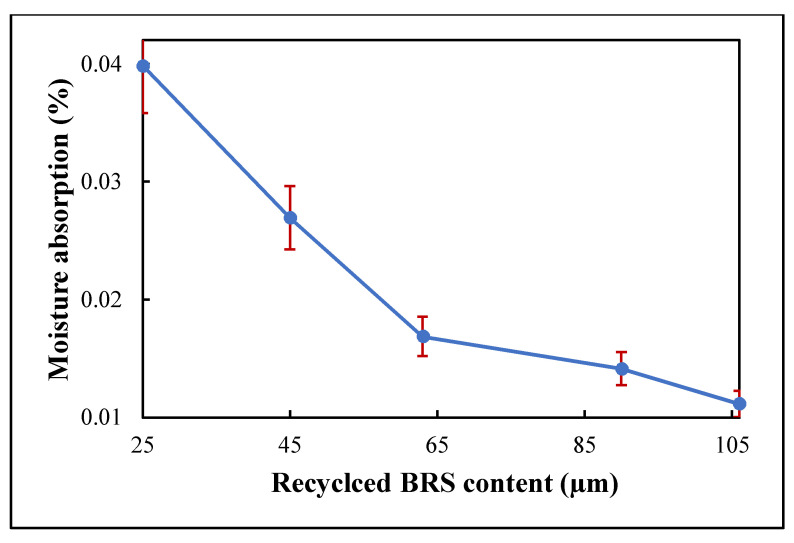
Variation of moisture absorption with filler size.

Moisture absorption significantly affects composite’s dielectric properties because water has a high dielectric constant and loss. It is reported that moisture absorption of <0.1% is ideal for electronic packaging applications [6,8,32]. Figure 5 shows the variation in the moisture absorption of PTFE/recycled BRS composites. It can be seen that the moisture absorption increases from 0.011% to 0.040% when the recycled BRS filler size is reduced from 106 μm to 25 μm. It is worth noting that the composite records moisture absorption lower than the ideal value recommended. The increase in moisture absorption is attributed to the higher surface area of the smaller-sized recycled BRS particles [8]. Furthermore, the deterioration of moisture absorption is related to the enhanced porosity and density in the composites [33].

### 3.3. Density

**Figure 6:**
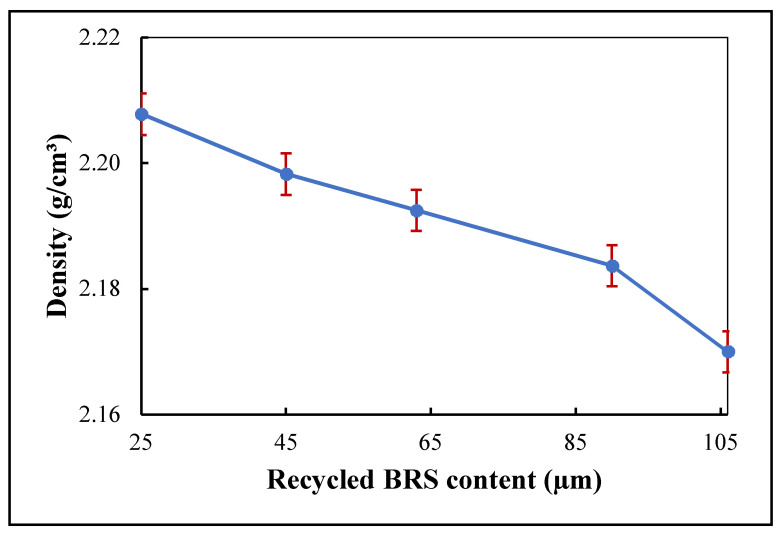
Variation of density with filler size.

The effect of recycled BRS filler size on the density of the PTFE matrix is shown in Figure 6. The 106 μm, 90 μm, 63 μm, 45 μm, 25 μm, recycled BRS composites had density values of 2.17, 2.18, 2.19, 2.20, and 2.21 g/cm^3^, respectively. Thus, decreasing recycled BRS particle size led to the increase in the density of the composites. A similar result has been reported by Jiang and Yuan [8]. The enhanced density is related to introducing a denser recycled BRS filler than the PTFE matrix [34]. In addition, smaller-sized particles possess more particles per unit volume than larger-sized particles. Therefore, the smaller-sized filler particles occupy less volume, leading to the increased density of the composites. The increase in the density is also due to the higher moisture absorbed by the composites [6,35]. This variation significantly affects the PTFE matrix’s CTE, tensile strength and dielectric properties [11].

### 3.4. Tensile Strength

**Figure 7:**
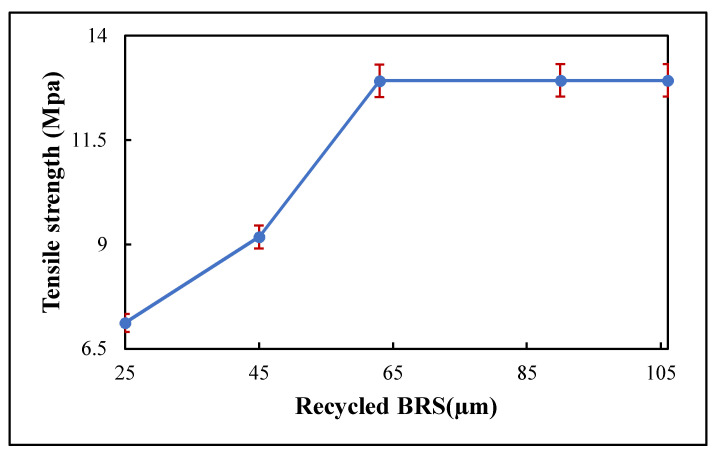
Variation of tensile strength with filler size.

The change of tensile strength as a function of recycled BRS particle size is presented in Figure 7. The 106 μm, 90 μm, 63 μm, 45 μm and 25 μm recycled BRS composites had respective tensile strength values of 12.93, 12.93, 12.92, 9.18 and 7.12 MPa. It could be seen that the reduction in particle size corresponded with a decrease in tensile strength consistent with the studies reported [36,37]. Although, the differences in tensile strength at 106 μm, 90 μm and 63 μm BRS sizes are smaller than at 45 μm and 25 μm filler sizes. This reduction in tensile strength is due to poor adhesion between the recycled BRS filler and PTFE matrix [36]. In addition, the smaller-sized particles with a higher surface area tend to absorb more water, which reduced the tensile strength of the PTFE matrix [38,39].

### 3.5. Coefficient of Thermal Expansion (CTE)

**Figure 8:**
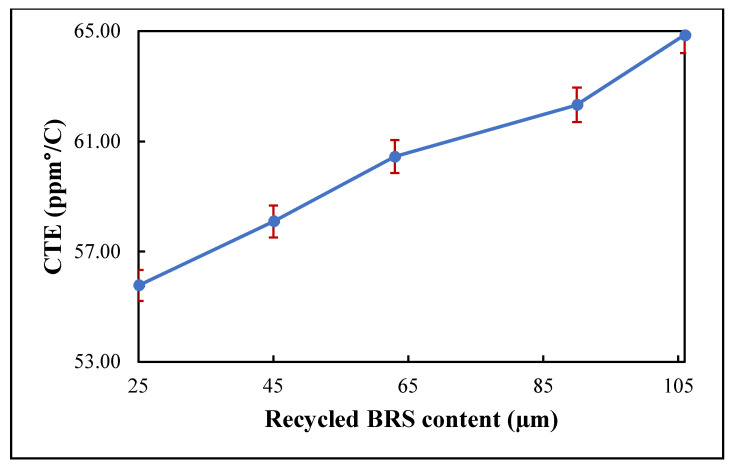
Variation of CTE with filler size.

The variation in CTE with recycled BRS particle size is shown in Figure 8. The composites showed a respective CTE of 64.8, 62.33, 60.45, 55.08 and 55.77 ppm/°C at 106 μm, 90 μm, 63 μm, 45 μm and 25 μm filler sizes. It is, therefore, evident that the decrease in filler size matched the drop in the CTE of the composites [8,36]. The variation is first attributed to the mismatch in the CTE of the PTFE matrix (~109 ppm/°C) and the recycled BRS filler (~4 ppm/°C [40,41]. In addition, smaller-sized filler particles have a larger surface area and higher density. Thus, the matrix volume decreases with smaller-sized particles, restricting the matrix expansion, which further reduces the CTE of the composites [12].

### 3.6. Complex Permittivity

**Figure 9:**
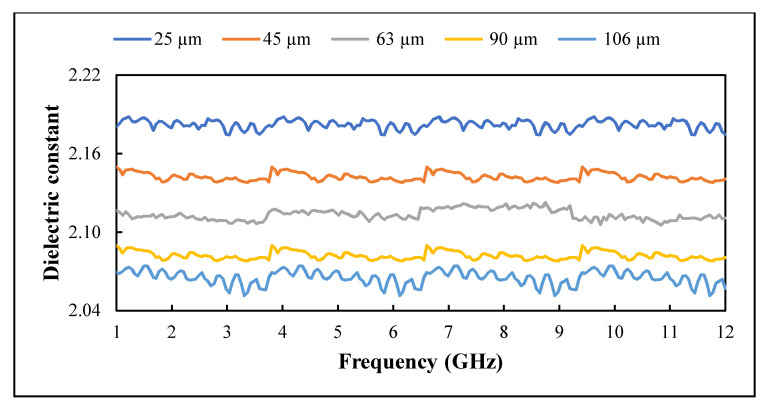
Variation of dielectric constant with filler size.

**Figure 10:**
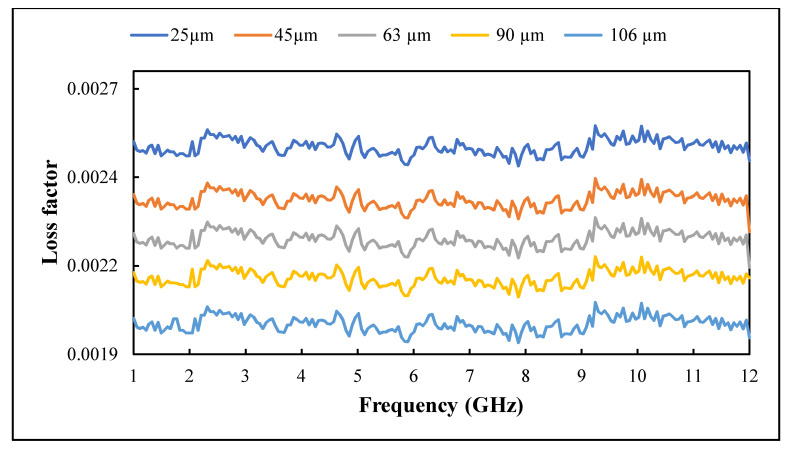
Variation of loss factor with filler size.

**Figure 11:**
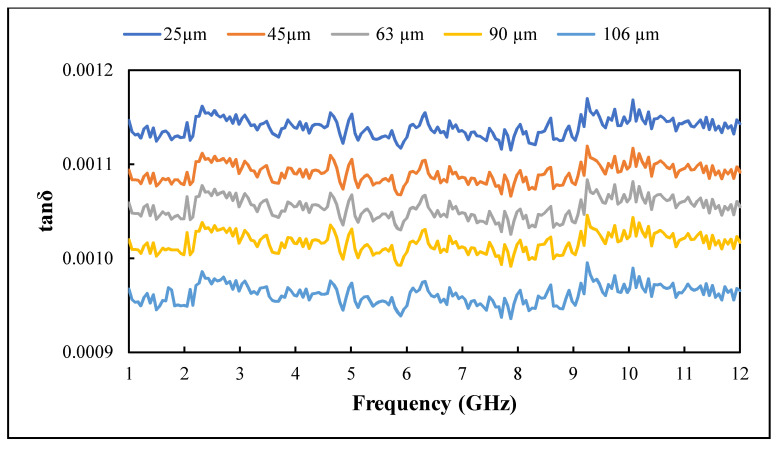
Variation of loss tangent with filler size.

The influence of recycled BRS filler size reduction on the dielectric constant and loss factor of PTFE/recycled BRS composites was studied. The variation of $\varepsilon^{\prime }$ and $\varepsilon^{\prime \prime }$ in the 1–12 GHz range is presented in Figure 9 and Figure 10, while the calculated tanδ is shown in Figure 11. It can be seen that the $\varepsilon^{\prime }$ and $\varepsilon^{\prime \prime }$ slightly decreased with the frequency [42,43,44]. In addition, the $\varepsilon^{\prime \prime }$ had a similar pattern for all composites, which is attributed to the calibration consistent with the loss factor result presented in [45]. The higher values of the $\varepsilon^{\prime }$ and $\varepsilon^{\prime \prime }$ at lower frequencies are due to the significant influence of charge relaxation and interfacial polarisation [46]. Generally, as frequency increases, the composite’s overall polarisation lags the alternating electric field. Thus, each polarisation process stops contributing, decreasing its dielectric constant and loss factor [47].

Further analysis showed that the $\varepsilon^{\prime }$ and $\varepsilon^{\prime \prime }$ of PTFE/recycled BRS composites increased with the reduction in recycled BRS filler size (Table 2), in agreement with previous work [12,36]. This behaviour is attributed to the higher densification and stronger interfacial polarisation [3]. Composites reinforced with smaller grain-sized particles tend to possess a more significant interfacial area, leading to extra interfacial polarisation, which increases the dielectric properties [12,48]. Moreover, at the same filler content, the number of particulates in the smaller-sized filler is higher than that in the bigger-sized filler. This occurrence leads to a denser composite, which increases the $\varepsilon^{\prime }$ and $\varepsilon^{\prime \prime }$ of the composite [12]. At 1 GHz, the values $\varepsilon^{\prime }$ and tanδ increased from 2.07 and 0.0010 to 2.18 and 0.0011 with a decreament of filler size from 106 μm to 25 μm. Additionally, the values of $\varepsilon^{\prime }$ and tanδ varied from 2.06 and 0.0010 to 2.17 and 0.0011 at 12 GHz.

### 3.7. Signal Transmission Speed

**Figure 12:**
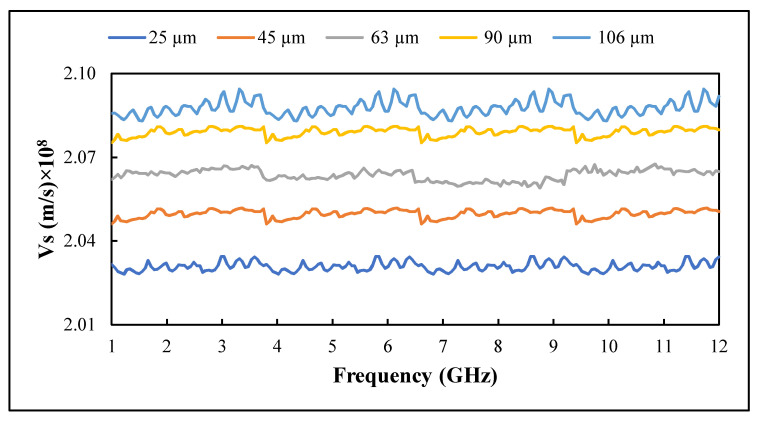
Variation of signal transmission speed with filler size.

The variation of signal transmission speed across the PTFE/recycled BRS composites at different recycled BRS sizes and frequencies is depicted in Figure 12. It can be seen that transmission speed decreases with filler size reduction. The higher transmission speed is associated with lower relative permittivity at larger filler sizes. At 1 GHz, PTFE/recycled BRS composites had Vs of 2.032 $\times \;10^{8}$ m/s, 2.046 $\times \;10^{8}$ m/s, 2.062 $\times \;10^{8}$ m/s, 2.075 $\times \;10^{8}$ m/s and 2.086 $\times \;10^{8}\;$m/s at 25 μm, 45 μm, 63 μm, 90 μm and 106 μm of recycled BRS filler sizes, respectively. The Vs increased to 2.034 $\times \;10^{8}\;$m/s, 2.050 $\times \;10^{8}$ m/s, 2.065 $\times \;10^{8}$m/s, 2.080 $\times \;10^{8}$ m/s and 2.092 $\times \;10^{8}$ m/s at 12 GHz for the same filler sizes.

The comparison of the PTFE/recycled BRS composite at a filler size of 63 μm with commercial high-frequency laminates is presented in Table 3. The laminates are PTFE-based materials produced by [49,50]. It can be seen that the PTFE/recycled BRS composite shows a lower dielectric constant, loss tangent, moisture absorption and CTE than the laminates. The highest tensile strength is achieved by the TLX-8 laminate, followed by the PTFE/recycled BRS composite. This result proves that recycled BRS glass can reinforce PTFE to produce a low-cost substrate for microwave applications.

## 4. Conclusions

The PTFE/recycled BRS composites were fabricated through the dry powder processing technique by varying the recycled BRS filler size. XRD profiles of the composites exhibited no unwanted peaks. The scanning electron microscope showed better dispersion of the filler at a larger recycled BRS size. EDX analysis indicated that no foreign element was present in the composites. The complex permittivity of PTFE/recycled BRS composites showed an increasing trend with recycled BRS filler size reduction. The moisture absorption and density of the composites also increased for the same reason. However, the tensile strength, CTE, and signal transmission speed decreased with recycled BRS filler size reduction. At 10 GHz, the 63 μm recycled BRS composite showed suitable dielectric properties ($\varepsilon^{\prime }=2.11$ and tanδ = 0.0011), CTE of 60.45 ppm/°C, low moisture absorption of 0.02% and favourable tensile strength of 12.92 MPa, ideal for microwave substrate applications.

## Figures and Tables

**Table 1:** Particle size distribution.

Representative Particle Size (μm)	Range of Particle Size (μm)
25	$X_{1}\;\leq \;25$
45	$25<X_{2}\leq \;45$
63	$45<X_{2}\leq \;63$
90	$63<X_{2}\leq \;90$
106	$90<X_{2}\leq \;106$

**Table 2:** Mean complex permittivity and loss tangent of PTFE/recycled BRS composites at different filler sizes.

Recycled BRS Size (μm)	$\varepsilon^{\prime }$	$\varepsilon^{\prime \prime }$	tanδ
25	2.18	0.0026	0.0011
45	2.14	0.0024	0.0011
63	2.11	0.0022	0.0011
90	2.08	0.0021	0.0010
106	2.07	0.0020	0.0010

**Table 3:** Comparison between PTFE/recycled BRS composite and commercial high-frequency laminates.

Name	$\varepsilon^{\prime }$	tanδ	Tensile Strength (MPa)	CTE (ppm/°C)	Moisture Absorption (%)	Reference
	At 10 GHz					
PTFE/recycled BRS composite	2.11 ± 0.05	0.0011 ± 0.00005	12.92 ± 0.005	60.45 ± 0.01	0.02 ± 0.00001	This study
AD250C	2.50	0.0013	6.00	196.00	0.04	[49]
AD255C	2.60	0.0013	8.1	196.00	0.03	[49]
TLX-8	2.55	0.0017	245	215.00	0.02	[50]

## Data Availability

Not applicable.
